# Dorland’s Medical Dictionary

**Published:** 1913-10-15

**Authors:** 


DORLAND'S MEDICAL DICTIONARY. A new and fully revised eition of this excellent work is just off the press of the W. B. Saunders Co. It is suiprising that we have so many excellent medical dictionaries and that they are so frequently revised or reprinted, and we are sometimes suspicious that the revisions are merely reprints. We have placed a high value on the former edition of Dorland and are most agreeably pleased to find this new edition to be a real revision containing a most carefull compilation of all the newer medical terms which we have had occasion to look up and others we note which are entirely new to us. The tremendous activity in medical science research and literary work is responsible for the constantly increasing medical and scientific vocabulary, and is the cause of the addition of nearly 5000 new words or combinations to be found in this new edition of Dorland. When we remember that some of our words have become obsolete and are eliminated from a properly edidet dictionary we get some idea of the value to one of an up to date dictionary. This dictionary is not only a well edited and designed dictionary, but it is a work of art from the publishers standpoint, it couldn't be better done so far as we can judge. It is a real joy to handle such a handy and yet convenient book. The definitions are terse and ample and the pronounciation of difficult terms is made so distinct that there is little excuse for one who owns this dictionary not correctly pronouncing all medical and scientific terms. It is not burdened with illustrations but some excellent colored plates and drawings add materially to its value where brief descriptions fail to convey adequate ideas. It is altogether an excellent book which every dentist would do well to have in his possession, and it will undoubtedly have a large sale to medical and dental students.

